# Efficacy and Safety of Cinobufacin Combined with Chemotherapy for Advanced Breast Cancer: A Systematic Review and Meta-Analysis

**DOI:** 10.1155/2020/4953539

**Published:** 2020-09-19

**Authors:** Jing Xu, Dongyun Li, Kexin Du, Jing Wang

**Affiliations:** ^1^Department of Hematology and Oncology, Dongzhimen Hospital Affiliated with Beijing University of Chinese Medicine, Beijing 100700, China; ^2^Beijing University of Chinese Medicine, Beijing 100029, China

## Abstract

**Background:**

Cinobufacin is a Chinese patent medicine widely used for breast cancer in China. However, no systematic review and meta-analysis have been published to validate its effects in breast cancer treatment. We, therefore, summarize the efficacy and safety of Cinobufacin combined with chemotherapy in order to provide rigid evidence for its clinical application.

**Methods:**

By searching multiple databases incepted to December 2019, the RCTs of breast cancer patients treated with Cinobufacin were screened according to the inclusion criteria, and the meta-analysis and sensitivity analysis were conducted using RevMan5.3.

**Results:**

A total of 1163 articles were retrieved, and 16 studies were included. The total sample size was 1331 cases, including 666 cases in the treatment group receiving Cinobufacin combined with chemotherapy and 665 cases in the control group receiving chemotherapy alone. Our study found that the ORR (overall response rate) (RR = 1.35, 95% CI: [1.23, 1.49], *P* < 0.00001), CBR (clinical benefit rate) (RR = 1.14, 95% CI: [1.08, 1.21], *P* < 0.00001), KPS scores (RR = 1.98, 95% CI: [1.45, 2.68], *P* < 0.0001), and pain relief rate (RR = 1.34, 95% CI: [1.01, 1.78] *P*=0.04 of the Cinobufacin combined with chemotherapy group were better than those of the chemotherapy group, and the difference was statistically significant. Our study also discovered that the tumor markers (CA125, CA153, and CEA) in the Cinobufacin combined with chemotherapy group were lower than those in the chemotherapy group, which heterogeneity was derived from the low-quality literature included in the study, but the results were robust. In addition, in terms of safety, we found that the incidences of gastrointestinal reactions (RR = 0.58, 95% CI: [0.48, 0.70], *P* < 0.00001), liver and kidney damage (RR = 0.57, 95% CI: [0.38, 0.84], *P*=0.004), and hair loss (RR = 0.61, 95% CI: [0.40, 0.92], *P*=0.02) in the Cinobufacin combined chemotherapy group were lower than those in the chemotherapy group, and the difference was statistically significant, but the incidences of peripheral neurotoxicity (RR = 0.69, 95% CI: [0.26, 1.85], *P*=0.46) and myelosuppression (RR = 0.78, 95% CI: [0.46, 1.34], *P*=0.37) in the combined group were similar to those of the chemotherapy group, and the difference was not statistically significant.

**Conclusions:**

Cinobufacin combined with chemotherapy can improve the clinical efficacy of breast cancer patients, enhance the quality of life of the patients, reduce the value of tumor markers such as CA125, CA153, and CEA, and lower the occurrence of adverse reactions such as gastrointestinal reactions, liver and kidney damage, and hair loss.

## 1. Introduction

Breast cancer is the malignant tumor with the highest female morbidity and the second highest mortality after lung cancer in the world. According to global cancer data statistics, there were more than 2 million new cases of breast cancer in 2018, accounting for 11.6% of the total number of new cancers. Among them, the incidence of breast cancer in China is as high as 36.1/100,000. More than 600,000 women die from breast cancer, accounting for 6.6% of total cancer-related deaths each year [1, 2]. Chemotherapy is one of the commonly used treatment methods for advanced breast cancer, but its application is limited because of its severe side effects, including gastrointestinal symptoms, myelosuppression, liver and kidney damage, etc [3].

Cinobufacin is a traditional Chinese patent medicine extracted from the skin of Bufo bufo gargarizans. Its components are toadoxin, dehydroxytoluotoxin, serotonin, amino acids, reducing sugar, arginine complex, etc. It has the functions of clearing away heat and detoxification, promoting blood circulation, and removing and resolving blood stasis [4]. In recent years, many studies have shown that Cinobufacin has antitumor effects, which may be related to its inhibition of tumor cell growth, induction of tumor cell apoptosis, and enhancement of immune function in the body [5]. Multiple meta-analyses have proved that Cinobufacin combined with chemotherapy can improve the efficacy and reduce adverse reactions in gastric cancer, liver cancer, non-small-cell lung cancer, rectal cancer, and other malignant tumors [6–9]. However, there are currently no evidence-based medicine data to demonstrate the efficacy and safety of Cinobufacin for breast cancer. Therefore, we carried out a meta-analysis of Cinobufacin based on the RCT literature of breast cancer to evaluate the efficacy and safety of its treatment and to provide guidance for the clinical application of Cinobufacin.

## 2. Methods

### 2.1. Protocol and Registration

First, the study was according to the statements of the Preferred Reporting Items for Systematic Reviews and Meta-Analyses (PRISMA) [10]. The protocol for this review has been registered on PROSPERO, and the registration number is CRD42020154411.

### 2.2. Literature Search

Our research retrieved the three major English databases of PubMed, Web of Science, Cochrane Library, and the four major Chinese databases of CNKI, WanFang, VIP, and SinoMed, with “Huachansu”, “cinobufotalin”, “cinobufacin”, “cinobufagin”, “cinobufatini”, “toad skin” and “breast cancer”, ”breast carcinoma”, and “breast tumor” as main inscriptions or keywords as well as subject words or free words. For example, the PubMed retrieval strategy was as follows: (Huachansu OR cinobufotalin OR cinobufacini OR cinobufagin OR cinobufatini OR toad skin) AND (breast cancer OR breast carcinoma OR breast tumor). The retrieval deadline is until December 2019. In addition, the references that were reviewed and included in the study were searched twice.

### 2.3. Inclusion and Exclusion Criteria

Our inclusion criteria include, first, randomized controlled trials published in China and abroad, regardless of language; second, all patients are confirmed as advanced breast cancer by pathology and imaging examination; third, the treatment group was treated with Cinobufacin combined with conventional chemotherapy, while the control group was treated with conventional chemotherapy; and fourth, inclusion of research outcome indicators is required (contains one of the following outcome indicators).

Our exclusion criteria include, first, no relevant outcome indicators; second, repeated publications, incomplete data, and second, repetitive, and incomplete data. It should be noted that incomplete data refer to per-protocol analysis instead of intention-to-treat analysis; and third, the intervention measures are Cinobufacin vs chemotherapy, or the control group is not chemotherapy alone.

The outcome indicators included in our study mainly involve clinical efficacy, KPS score, pain relief rate, tumor markers (CA125, CA153, and CEA), and adverse reactions (gastrointestinal reaction, myelosuppression, alopecia, liver and kidney damage, and peripheral neurotoxicity). The clinical efficacy includes overall response rate (ORR) and clinical benefit rate (CBR)—ORR = CR + PR/total cases × 100% and CBR = CR + PR + SD/total cases × 100% [11].

### 2.4. Data Extraction

We first use EndNote to search and remove duplicate documents and read the remaining documents in depth. Two researchers (JX and KXD) independently screened the literature, extracted data, and cross-checked according to the inclusion and exclusion criteria. For those in doubt, they will be discussed or decided by a third researcher (DYL). The data extracted by this research include first author, year of publication, age, number of cases in each group, intervention measures, course of treatment, outcome indicators, etc.

### 2.5. Quality Evaluation

Two researchers (KXD and JW) used the risk bias assessment tool in the Cochrane evaluation manual [12] to evaluate the quality of the included literature and then cross-checked it. The evaluation criteria include random sequence generation, allocation concealment, blinding participants and personnel, blinding of outcome assessment, incomplete outcome data, selective reporting, and other bias. Disagreements are resolved through discussion or consultation with a third evaluator (DYL).

### 2.6. Statistical Analysis

We used RevMan5.3 software to conduct meta-analysis, heterogeneity test, sensitivity analysis and publication bias test on the included studies. The significance level is set to *α* = 0.05, and the heterogeneity is quantitatively analyzed by *I*^2^. If *P* < 0.05, *I*^2^ ≥ 50%, there is obvious heterogeneity between the results of each study, and the random effect model is used for analysis. Mean difference (MD) is used for those with the same measurement unit, and relative risk (RR) and 95% confidence interval (95% CI) are used for binary classification variables. If the clinical heterogeneity is obvious, then the subgroup analysis or sensitivity analysis should be used for treatment.

## 3. Results

### 3.1. Search Results

**Figure 1:**
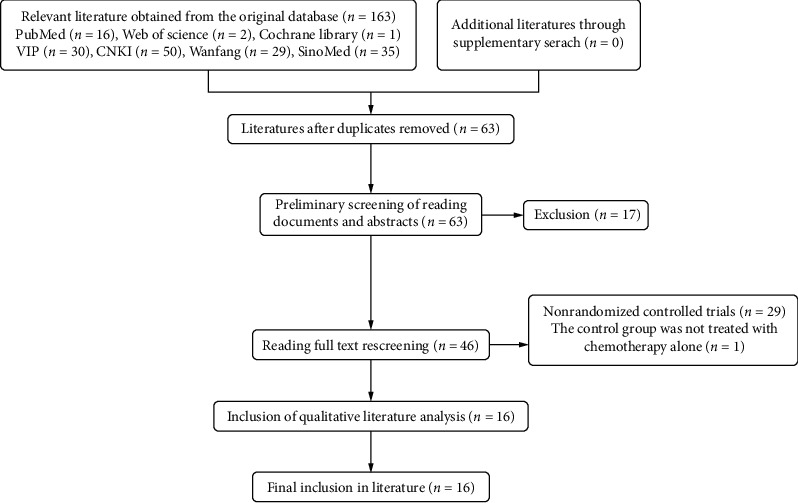
Flowchart of literature screening.

We initially retrieved 163 related literature studies, and through reading the title, abstract, and full text of the literature studies, excluding animal experiments, repeated studies, and reviews, we finally met the inclusion criteria of 16 RCTs (Figure 1).

### 3.2. Basic Characteristics of Included Studies

The 16 literature studies [13–28] included in our study included 1331 patients, of which 666 patients in the Cinobufacin combined ith chemotherapy group and 665 patients in the chemotherapy-alone group. All the subjects were adult women, and the baseline of each study was comparable. The time span included in the study was 18 years. It should be pointed out that there are no randomized controlled trials of Cinobufacin in breast cancer treatment in foreign literature. The related studies Cinobufacin and breast cancer are reviews or related mechanism studies (Table 1).

### 3.3. Quality Evaluation of Included Studies

**Figure 2:**
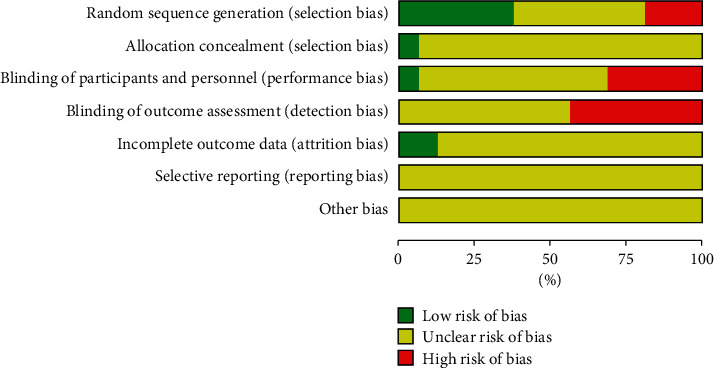
The diagram of risk of bias in included studies.

Nine of the 16 studies in our study reported specific random sequence generation methods, among which three studies [17, 18, 21] were grouped according to the order of admission or time and were rated as high-risk bias, while six studies [13, 16, 20, 26–28] were rated as low-risk bias, and the remaining seven studies only mentioned random and did not report the implementation of a specific random scheme. One study [20] was randomly grouped using the sealed envelope method, and no studies reported the implementation of the blind method. None of the studies reported the concealment of random allocation. Sixteen studies did not fully report the predetermined indicators, and there were cases where the results were selectively reported (Figure 2).

### 3.4. Meta-Analysis Results

#### 3.4.1. Clinical Efficacy

**Figure 3:**
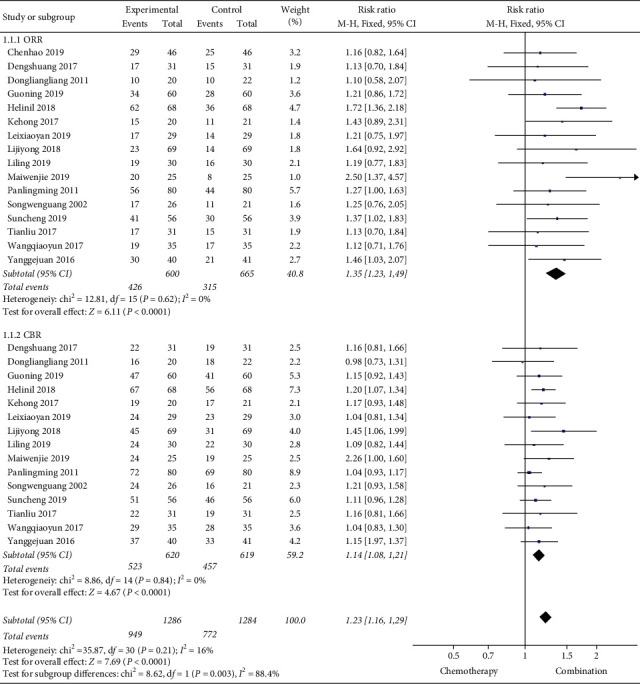
A meta-analysis of chemotherapy combined with Cinobufacin and chemotherapy alone for clinical efficacy.

The clinical efficacy of our research includes ORR (overall response rate) and CBR (clinical benefit rate). The 16 studies [13–28] (1331 cases) we included all reported ORR. The meta-analysis results found that the ORR of Cinobufacin combined with chemotherapy was superior to simple chemotherapy (RR = 1.35, 95% CI: [1.23, 1.49], *P* < 0.00001). We included 15 studies [14–28] (1239 cases) reported CBR. The results of meta-analysis showed that the CBR of Cinobufacin combined with chemotherapy was better than that of chemotherapy alone (RR = 1.14, 95% CI: [1.08, 1.21], *P* < 0.00001) (Figure 3).

#### 3.4.2. KPS Score and Pain Relief Rate

**Figure 4:**
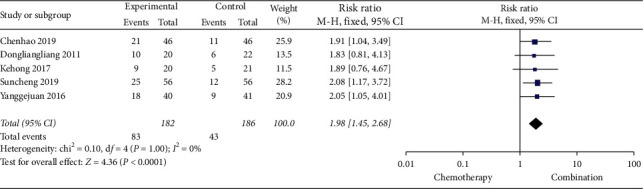
A meta-analysis of chemotherapy combined with Cinobufacin and chemotherapy alone for KPS scores after treatment.

**Figure 5:**
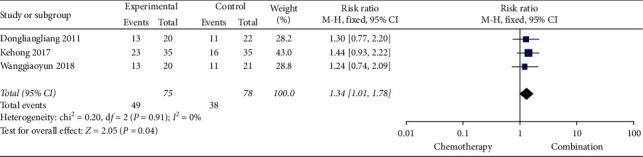
A meta-analysis of chemotherapy combined with Cinobufacin and chemotherapy alone for pain relief rate.

Five studies [13, 19, 21, 22, 25] (368 cases) reported KPS scores, and three studies [17, 19, 22] (153 cases) reported pain relief rate. The results of meta-analysis showed that the KPS score and pain relief rate of the Cinobufacin combined with chemotherapy group were better than those of chemotherapy-alone group, the RR of KPS scores was 1.98, 95% CI was 1.45 to 2.68, *P*-value was less than 0.0001, and the RR of pain relief rate was 1.34, 95% CI was 1.01 to 1.78, *P*=0.04 (Figures 4 and 5).

#### 3.4.3. Tumor Markers

**Figure 6:**
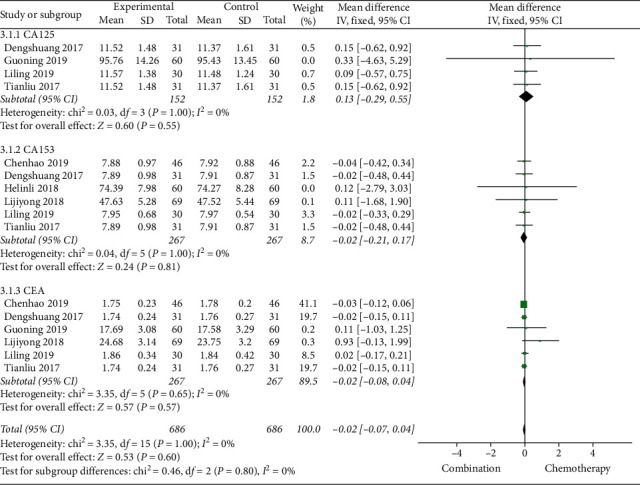
A meta-analysis of chemotherapy combined with Cinobufacin capsules and chemotherapy alone in tumor markers (CA125, CA153, and CEA) before treatment.

**Figure 7:**
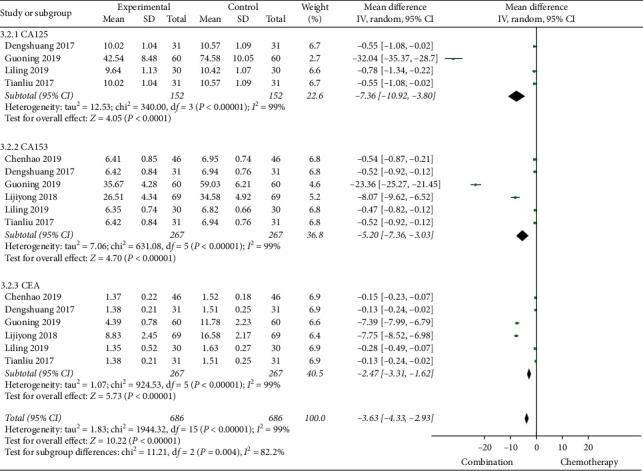
A meta-analysis of chemotherapy combined with Cinobufacin capsules and chemotherapy alone in tumor markers (CA125, CA153, and CEA) after treatment.

The six studies [13, 14, 18, 20, 26, 28] were reported the changes of CA153 and CEA, of which 4 studies [14, 18, 20, 26] reported changes in CA125. Through a comparative analysis of tumor markers before and after treatment in 1372 patients, we found that the heterogeneity between CA125, CA153, and CEA groups before treatment was relatively small and the fixed effects model was selected for meta-analysis. The results showed that there was no statistical difference in tumor markers before treatment (*P* > 0.05) (Figure 6). After treatment, the heterogeneity among CA125, CA153, and CEA groups was large and the random effects model was selected for meta-analysis. The results showed that the tumor markers of Cinobufacin combined with chemotherapy after treatment were lower than those of chemotherapy alone, and the difference was statistically significant; the MD of CA125 was −7.36, 95% CI was −10.92 to −3.80, *P*-value was less than 0.0001, the MD of CA153 was −5.20, 95% CI was −7.36 to −3.03, *P*-value was less than 0.00001, and the MD of CEA was −2.47, 95% CI was −3.31 to −1.62, *P*-value was less than 0.00001 (Figure 7).

#### 3.4.4. Adverse Reactions

**Figure 8:**
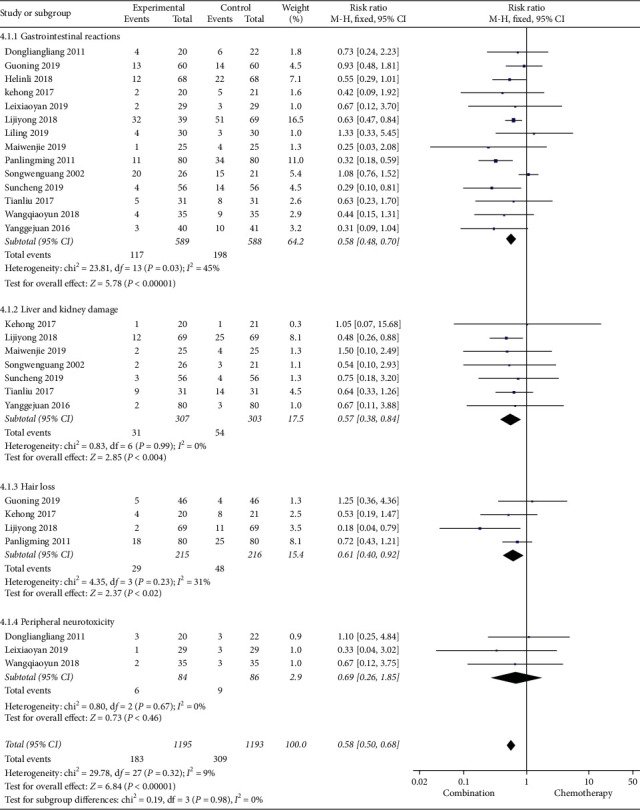
A meta-analysis of chemotherapy combined with Cinobufacin and chemotherapy alone for adverse reactions.

**Figure 9:**
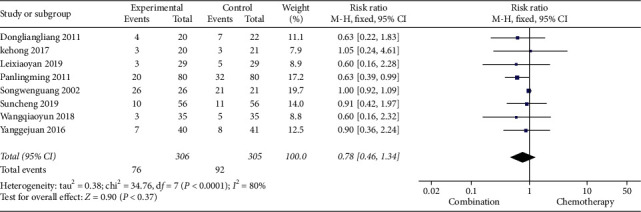
A meta-analysis of chemotherapy combined with Cinobufacin and chemotherapy alone in myelosuppression.

14 studies reported adverse reactions, including gastrointestinal reactions, liver and kidney damage, hair loss, peripheral neurotoxicity, bone marrow suppression, and so on. According to the heterogeneity test, the heterogeneity of gastrointestinal reaction, liver and kidney damage, hair loss, and peripheral neurotoxicity was small, and hence the fixed effects model was used for meta-analysis, while the heterogeneity of bone marrow suppression study was large, and hence the random effects model was used for meta-analysis. The results of meta-analysis showed that the incidences of gastrointestinal reaction, liver and kidney damage, and hair loss in the Cinobufacin combined with chemotherapy group were lower than those in the chemotherapy-alone group, and the RR of gastrointestinal reaction was 0.58, 95% CI was 0.48 to 0.70, *P*-value was less than 0.00001, the RR of liver and kidney damage was 0.57, 95% CI was 0.38 to 0.84, *P*=0.004, and the RR of hair loss was 0.61, 95% CI was 0.40 to 0.92, *P*=0.02. The incidences of peripheral neurotoxicity and myelosuppression in the combination group were similar to those in the chemotherapy group, and the difference was not statistically significant, the RR of peripheral neurotoxicity was 0.69, 95% CI was 0.26 to 1.85, *P*-value was 0.46, and the RR of myelosuppression was 0.78, 95% CI was 0.46 to 1.34, *P*=0.37 (Figures 8 and 9).

### 3.5. Sensitivity Analysis

**Figure 10:**
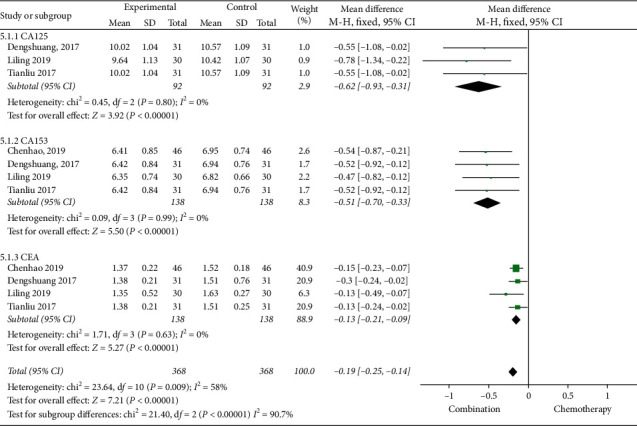
A meta-analysis of sensitivity analysis of chemotherapy combined with Cinobufacin and chemotherapy alone in tumor markers (CA125, CA153, and CEA) after treatment.

Our results showed that the heterogeneity of tumor markers (CA125, CA153, and CEA) after treatment was relatively large (*I*^2^ = 99%). After the comparative analysis was included in the literature, the heterogeneity of tumor markers after treatment was significantly reduced (*I*^2^ = 0) after the removal of the studies by Guo et al. [14] and Li [28], so we considered that the heterogeneity of tumor markers after treatment was mainly related to the quality of the included study (Figure 10).

### 3.6. Publication Bias Analysis

**Figure 11:**
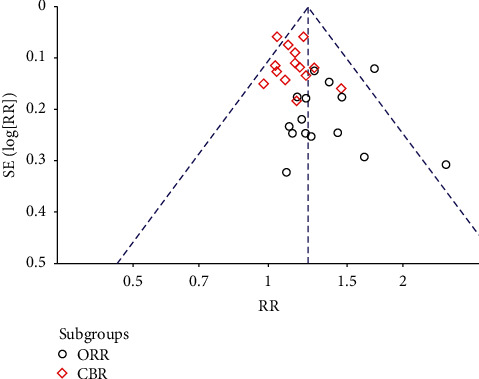
Funnel chart of clinical efficacy.

The funnel chart analysis of clinical efficacy showed that the results were not completely symmetrical, which was related to the low quality of the study and the small sample size (Figure 11).

## 4. Discussion

In this study, the meta-analysis method was used to merge and analyze 16 randomized controlled literature studies on the efficacy and safety of Cinobufacin combined with chemotherapy for breast cancer. The results of meta-analysis showed that the ORR, CBR, KPS scores, and pain relief rate of the Cinobufacin combined with chemotherapy group were better than those of the chemotherapy-alone group, which suggested that Cinobufacin combined with chemotherapy could improve the clinical efficacy and quality of life of the patients with breast cancer. Our study also found that the tumor markers (CA125, CA153, and CEA) of the Cinobufacin combined with chemotherapy group were lower than those of the chemotherapy-alone group. The heterogeneity was related to the low-quality literature of the included studies, but the results were stable. In terms of safety, the incidences of gastrointestinal reactions, liver and kidney damage, and hair loss in the Cinobufacin combined chemotherapy group were lower than those in the simple chemotherapy group, and the difference was statistically significant, but the incidences of peripheral neurotoxicity and myelosuppression in the combined group were similar to those in the chemotherapy group, and the difference was not statistically significant.

Chemotherapy is the most common and direct clinical treatment for advanced breast cancer, but it usually requires large doses of two or more chemotherapeutic agents, which will bring corresponding side effects when reaching the treatment dose. The minor side effects will have a certain impact on the daily life of the patients, and the severe toxicities will threaten the physical and mental health of the patients, which will lead to the failure of chemotherapy and affect the prognosis [29–31]. Therefore, the combination of traditional Chinese and Western medicine has become a common clinical treatment for malignant tumors [32]. As the incidence of breast cancer increases year by year, an antitumor traditional Chinese medicine preparation independently developed by China, Cinobufacin, can be used in the field of breast cancer in combination with the chemotherapeutics to reduce the adverse reactions of patients and improve the quality of life of patients [33]. The mechanism may be as follows: First, apoptosis of human breast cancer cell line T-47D can be induced by increasing the expression level of caspase-3 [34]; second, apoptosis of MDA-MB-231 can be induced by destroying the cytoskeleton, resulting in abnormal changes of the cell surface ultrastructure and morphology [35]; Third, it can inhibit the growth and proliferation of human breast cancer cell lines MDA-MB-468 and BT549 by inhibiting their proliferation and migration and the activity of PI3K/Akt signaling pathway [36, 37]; Fourth, many studies have shown that due to a large number of fibrin accumulation and platelet aggregation around cancer cells, patients with malignant tumors are prone to coagulation dysfunction, which makes the blood present a hypercoagulable state, while traditional Chinese medicine for promoting blood circulation and removing blood stasis can expose cancer cells, so they are more vulnerable to the attack of chemotherapy drugs [38, 39]. Therefore, the efficacy of Cinobufacin in clearing away heat and detoxification, promoting blood circulation, and removing stasis can play a role of “increasing efficiency and reducing toxicity” when combined with chemotherapy.

At present, a large number of experimental studies have found that Cinobufacin has antitumor effects on breast cancer, lung cancer, esophageal cancer, gastric cancer, liver cancer, bladder cancer, etc. [40]. Ni et al. [41] treated MGC-803 and BGC-823 GC cells with different concentrations of Cinobufacin and found that Cinobufacin has significant antitumor cell proliferation and apoptosis effects both in vivo and in vitro and can inhibit the growth of gastric cancer cells by inhibiting the Akt/mTOR pathway and induce cell apoptosis through the internal pathway. Yin et al. [42] established a nude mouse xenograft model and found that Cinobufacin can inhibit the growth of liver metastases by reducing the expression of MMP-2, MMP-9 and VEGF. Yang et al. [43] also found that Cinobufacin can inhibit the growth of human bladder cancer cells in vivo and in vitro through Fas/Fasl and TNF-*α*/TNFR1 pathways. Not only experimental studies have confirmed the scientific antitumor effect of Cinobufacin but also a large number of clinical studies have confirmed the rationality of its clinical application. Sha et al. [44] through the RCT test on the effect of Cinobufacin capsule combined with raltitrexed and oxaliplatin on advanced colorectal cancer found that Cinobufacin capsule combined with raltitrexed and oxaliplatin can enhance the immune function of patients with advanced colorectal cancer, reduce tumor markers, and inhibit the growth and metastasis of tumor cells and neovascularization. Wang et al. [45] found that patients with advanced liver cancer who were treated with Cinobufacin as a single agent had a lower rate of disease deterioration (11.4%) and a higher total effective rate after treatment (82.86%). Serum total bilirubin and alanine aminotransferase decreased significantly.

Our study also found that Cinobufacin combined with chemotherapy can reduce the incidences of gastrointestinal reactions, liver and kidney damage, and alopecia in breast cancer patients, and there are also relevant clinical reports. Dong [46] found that the incidences of nausea, vomiting, and leukopenia were lower in the combined group than in the control group after randomly dividing 68 patients with advanced colon cancer into the chemotherapy group and chemotherapy combined with Cinobufacin group. Cao, et al. [47] found that the combined group could reduce the incidence of myelosuppression and improve the disease control rate through the RCT trial of Cinobufacin injection combined with first-line chemotherapy in the treatment of advanced non-small-cell lung cancer. In addition, studies on adverse reactions related to Cinobufacin itself have mainly focused on reports of Cinobufacin injection. Cheng [48] retrospectively analyzed 272 cases of adverse reactions/events of Cinobufacin injection and found that the adverse reactions produced by Cinobufacin injection were mainly rapid-onset type, which mainly manifested as rapid-onset skin reactions in addition to venous injury and other adverse reactions. Zhang [49] analyzed 60 cases of adverse reactions caused by Cinobufacin injection and found that the main reactions were allergic and febrile reactions, and no deaths were observed.

Our study is a comprehensive analysis of the RCT literature of Cinobufacin for breast cancer, but there are still some deficiencies. First, the chemotherapy methods used in the studies included in this study are not the same. Although they are all patients with advanced breast cancer, the pathological stage is not exactly the same, which increases the clinical heterogeneity of the study.

Second, blind methods and random allocation concealment were not implemented in some of the included studies, which made the quality of the included studies low. Third, although the Chinese and English databases were searched extensively, the included cases were still from China. Finally, this article has not yet carried out stratified analysis of different dosage forms of Cinobufacin, which may increase the heterogeneity of the study. By consulting the relevant literature, we found that the main components of the three formulations were dried toad skin extract, which could play an antitumor role by inhibiting the growth and reproduction of tumor cells, inducing cell apoptosis and antimetastasis, targeting Na^+^/K^+^-ATPase activity, inhibiting tumor angiogenesis, and inhibiting the steroid receptor coactivator family [50].

## 5. Conclusion

In summary, the results of this study indicate that Cinobufacin combined with chemotherapy can improve the clinical efficacy of breast cancer patients, enhance the quality of life of the patients, reduce the value of tumor markers such as CA125, CA153, and CEA, and lower the occurrence of adverse reactions such as gastrointestinal reactions, liver and kidney damage, and hair loss. However, the conclusion of safety should be used carefully especially, the corresponding adverse reactions caused by different dosage forms of Cinobufacini itself should be considered. In future, we will carry out more clinical studies on different dosage forms of Cinobufacin and further compare the efficacy of different dosage forms and the same chemotherapy regimen in the treatment of breast cancer.

## Figures and Tables

**Table 1:** Basic characteristics of 16 included studies.

Author and Year	Treatment Course (weeks)	Age (T/C)	Number(T/C)	Interventions (T/C)	Outcome Indicators
Chen 2019 [13]	6w	46.34 ± 7.88/45.67 ± 7.91	46/46	Cinobufacin + NX/NX	A1C2C3

Guo 2019 [14]	8w	45.37 ± 7.68/45.54 ± 7.82	60/60	Cinobufacin + pirarubicin/Pirarubicin	A1A2C1C2C3D1D4

Lei 2019 [15]	6w	50.42 ± 3.62/50.46 ± 3.68	29/29	Cinobufacin + DC/DC	A1A2D1D2D5

He 2018 [16]	8w	43.7 ± 1. 9/44.33 ± 2.1	68/68	Cinobufacin + TEC/TEC	A1A2D1

Wang 2018 [17]	6w	Median age 54	35/35	Cinobufacin + DC/DC	A1A2B2D1D2D5

Tian 2017 [18]	9–18w	50.27 ± 6.23/49.57 ± 5.86	31/31	Cinobufacin + basic scheme containing capecitabine/Basic scheme containing capecitabine	A1A2C1C2C3

Ke 2017 [19]	12w	38. 3 ± 8. 9/39. 5 ± 8.8	20/21	Cinobufacin + CAF/CAF	A1A2B1B2D1D3D4

Deng 2017 [20]	6w	50.27 ± 6.23/49.57 ± 5.86	31/31	Cinobufacin + basic scheme containing capecitabine/Basic scheme containing capecitabine	A1A2C1C2C3

Yang 2016 [21]	8w	34.58 ± 14.44	40/41	Cinobufacin + TAC/TAC	A1A2B1D1D2D3

Dong 2011 [22]	6w	Average age 53	20/22	Cinobufacin + DC/DC	A1A2B1B2D1D2D5

Pan 2011 [23]	6w	Average age 56	80/80	Cinobufacin + CAF/CAF	A1A2D1D2D4

Song 2002 [24]	4w	Median age 54	26/21	Cinobufacin + CAF/CAF	A1A2D1D2D3

Sun 2019 [25]	6w	41.85 ± 2.25/43.56 ± 3.02	56/56	Cinobufacin + TEC/TEC	A1A2B1D1D2D3

Li 2019 [26]	9w	49.34 ± 7.34/48.36 ± 8.52	30/30	Cinobufacin + Basic scheme containing capecitabine/Basic scheme containing capecitabine	A1A2C1C2C3D1

Mai 2019 [27]	6w	44.10 ± 2.92/43.50 ± 3.71	25/25	Cinobufacin + docetaxel sequential CEF/Docetaxel sequential CEF	A1A2D1D3

Li 2018 [28]	—	47.5 ± 11.2/48.5 ± 10.8	69/69	Cinobufacin + pemetrexed combined with DDP/Pemetrexed combined with DDP	A1A2C2C3D1D3D4

Notes: T/C: treatment group/control group. A1(ORR); A2(CBR); B1(KPS score); B2(pain relief rate); C1(CA125); C2(CA153); C3(CEA); D1(gastrointestinal reaction); D2(myelosuppression); D3(liver and kidney damage); D4(alopecia); D5(peripheral neurotoxicity). TAC: docetaxel + pirarubicin + cyclophosphamide; TEC: docetaxel + epirubicin + cyclophosphamide; NX: capecitabine + vinorelbine; CAF: cyclophosphamide + pirarubicin + 5-FU; DC: docetaxel + capecitabine; CEF: cyclophosphamide + epirubicin + 5-FU.

## Data Availability

All the data were taken from the published studies.

## Abbreviations

CNKI: Chinese National Knowledge Infrastructure
RCT: randomized controlled trials
ORR: overall response rate
CBR: clinical benefit rate
KPS: Karnofsky Performance Status
CR: complete response
PR: partial response
SD: stable disease
RR: relative risk
CI: confidence interval
MD: mean difference.
